# Exploring genetic variation for salinity tolerance in chickpea using image-based phenotyping

**DOI:** 10.1038/s41598-017-01211-7

**Published:** 2017-05-02

**Authors:** Judith Atieno, Yongle Li, Peter Langridge, Kate Dowling, Chris Brien, Bettina Berger, Rajeev K. Varshney, Tim Sutton

**Affiliations:** 1 Australian Centre for Plant Functional Genomics, School of Agriculture, Food and Wine, University of Adelaide, Waite Campus, PMB 1, Glen Osmond, SA 5064 Australia; 2 School of Agriculture, Food and Wine, University of Adelaide, Waite Campus, PMB1, Glen Osmond, SA 5064 Australia; 3 The Plant Accelerator, School of Agriculture, Food and Wine, University of Adelaide, Waite Campus, PMB1, Glen Osmond, SA 5064 Australia; 4 Centre of Excellence in Genomics, International Crops Research Institute for the Semi-Arid Tropics (ICRISAT), Patancheru, India; 5 South Australian Research and Development Institute, GPO Box 397 Adelaide, South Australia, 5001 Australia; 6 School of Agriculture, Food and Wine, University of Adelaide, Waite Campus, PMB1, Glen Osmond, SA 5064 Australia

## Abstract

Soil salinity results in reduced productivity in chickpea. However, breeding for salinity tolerance is challenging because of limited knowledge of the key traits affecting performance under elevated salt and the difficulty of high-throughput phenotyping for large, diverse germplasm collections. This study utilised image-based phenotyping to study genetic variation in chickpea for salinity tolerance in 245 diverse accessions. On average salinity reduced plant growth rate (obtained from tracking leaf expansion through time) by 20%, plant height by 15% and shoot biomass by 28%. Additionally, salinity induced pod abortion and inhibited pod filling, which consequently reduced seed number and seed yield by 16% and 32%, respectively. Importantly, moderate to strong correlation was observed for different traits measured between glasshouse and two field sites indicating that the glasshouse assays are relevant to field performance. Using image-based phenotyping, we measured plant growth rate under salinity and subsequently elucidated the role of shoot ion independent stress (resulting from hydraulic resistance and osmotic stress) in chickpea. Broad genetic variation for salinity tolerance was observed in the diversity panel with seed number being the major determinant for salinity tolerance measured as yield. This study proposes seed number as a selection trait in breeding salt tolerant chickpea cultivars.

## Introduction

Chickpea (*Cicer arietinum* L.) is an important legume crop used as human food, animal feed and is also grown in rotation with cereal crops to fix nitrogen in the soil and to act as a disease break^1^. Chickpea is generally grown in semi-arid regions which can be prone to soil salinity but it is considered to be very sensitive to salinity with an estimated global annual chickpea yield loss of between 8–10% attributed to salinity^2^. Salinity impacts negatively on both the vegetative^3–5^ and reproductive growth stages^6–9^, with the reproductive stage the more salt sensitive^10^. The above studies show salinity has an adverse effect on shoot biomass, podding, and pod filling in chickpea.

Salinity limits plant growth and development through both shoot ion independent and shoot ion dependent stresses^11, 12^. Shoot ion independent stress immediately follows salinity stress, whereas ionic stress manifests after several days or weeks following exposure to salt, once ions accumulate in the shoot^11, 12^. Shoot ion independent stress results from hydraulic resistance imposed by NaCl in the plant xylem^13^ as well as the reduction in external osmotic potential (osmotic stress) which interferes with water uptake leading to a reduction in plant growth rate^14–16^. Such a reduction in growth rate due to salinity must ultimately translate to a reduction in shoot biomass. Turner *et al*.^6^ and Vadez *et al*.^17^ found that salt tolerant chickpea genotypes (measured as seed yield under low to medium salinity) are able to maintain high shoot biomass under salinity.

Many plants species, including chickpea, can tolerate osmotic stress by producing metabolites for osmotic adjustment^11^. Recently, a study conducted by Dias *et al.* ^18^ established differential accumulation of metabolites involved in the TCA cycle, carbon and amino acid metabolism in two chickpea genotypes (Genesis 836 & Rupali) that have been shown to contrast in salinity tolerance^4^. Rupali was found to have increased levels of amino acids, sugars and organic acids from TCA cycle compared to Genesis 836 following salinity treatment^18^. Production of these metabolites would be energy demanding which explains reduction in growth in Rupali when exposed to salinity. Prolonged exposure of plants to salinity causes Na^+^ and Cl^−^ to accumulate in plant tissues to toxic levels leading to plant death manifested by leaf senescence and necrosis^11, 19–21^. To protect the photosynthetic apparatus in young developing leaves from ion toxicity, plants exclude sodium from the transpiration stream by regulating as best as possible sodium net uptake and sequestering ions in the root cell vacuoles. Ions in the transpiration stream which enters the shoot can be sequestered in the lower, older leaves^11^. Screening diverse germplasm of chickpea for salinity tolerance revealed a wide spectrum of senescence displayed by different chickpea genotypes under salinity stress^22^, which demonstrated that different chickpea genotypes have varying levels of ion exclusion or tissue tolerance. The contribution of ions to salt sensitivity in chickpea has recently gained interest, with Na^+^ rather than Cl^−^ found to be toxic^6, 8, 23^. Vadez *et al*.^7^ did not find an association between salinity tolerance (seed yield per plant in saline soil) and accumulation of Na^+^ in total vegetative biomass at 50 days after sowing (DAS) in a germplasm collection of chickpea, whereas, Turner *et al*.^6^ established a negative correlation between Na^+^ accumulation in the youngest fully expanded leaf at 98 DAS with salinity tolerance (seed yield under 40 mM NaCl) in 55 chickpea genotypes. These differences could be attributed to different sampling strategies for leaf tissues (different time points and developmental stage) employed in the two studies.

Genetic variation within cultivated chickpea (*Cicer arietinum*) and related species can be exploited to improve salinity tolerance in future varieties. Previous studies on limited numbers of chickpea genotypes suggested the availability of limited genetic variation for salinity tolerance in chickpea^24, 25^. However, more recent research to explore variation in chickpea germplasm collections has demonstrated a broad range of genetic variation for salinity tolerance, such as that represented in the chickpea Reference Set^6, 7, 9^. Formed to enable efficient utilisation of chickpea genetic resources, the Reference Set is composed of geographically diverse material that includes; 267 landraces, 13 advanced lines and cultivars, 7 wild *Cicer* accessions and 13 accessions whose classification is unknown^26^. Characterisation of the Reference Set using 50 SSR markers revealed that it is rich in allelic diversity^26^ and can be mined for genetic variation of value in breeding.

The rapid development of new, high-resolution and high-throughput phenotyping technologies in plant science has provided the opportunity to more deeply explore genetic variation for salinity tolerance in crop species and identify traits that are potentially novel and relevant to yield improvement. Vadez *et al*.^27^ utilised a high-throughput, 3D scanning technique to monitor leaf area development in relation to plant water use in cowpea and peanut. Several studies in cereals have used high-throughput phenotyping technology under controlled environmental conditions to gain a better understanding of the physiological processes associated with salinity stress^20, 21, 28–32^. In contrast, similar studies examining salinity response in legume species have not been reported. Salinity response, measured as effect of salt on growth rate at different developmental times, could explain genotypic variation for salinity tolerance in chickpea. To investigate this hypothesis, we have utilised an image-based phenotyping platform to enable quantitative, non-destructive assessment of temporal responses of chickpea to salinity and we relate these responses to seed yield under saline conditions. This has allowed investigation into the complex relationship between different traits, with the aim of identifying novel traits that can be applied as selection tools in breeding programs.

## Results

**Figure 1:**
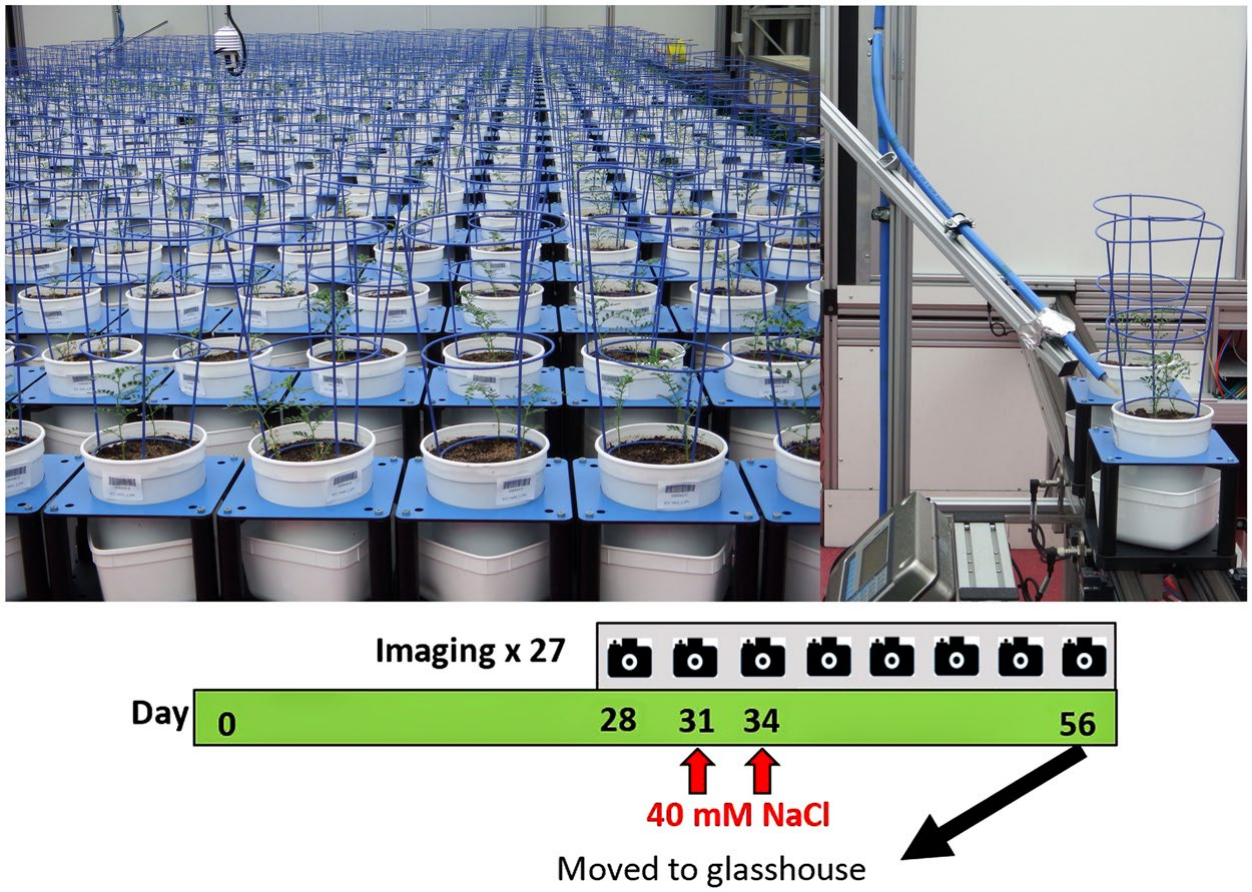
Salinity tolerance phenotyping in The Plant Accelerator. Plants were imaged at 28 DAS for 3 consecutive days prior to 40 mM NaCl application in two increments over 2 days. Plants were daily imaged until 56 DAS. Right pane shows 6-week-old chickpea plants on conveyor belts leaving the imaging hall proceeding to an automatic weighing and watering station.

**Figure 2:**
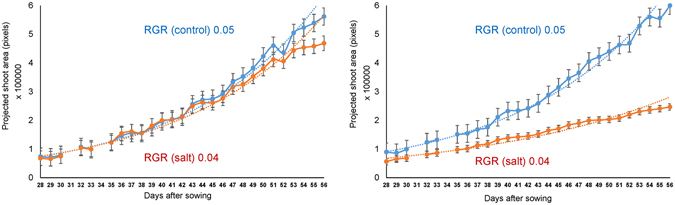
Non-destructive imaging of chickpea plants over time under 0 and 40 mM NaCl. Growth rates of ICC 95 (salt tolerant) and ICC 2720 (salt sensitive) under 0 mM NaCl (control) and 40 mM NaCl (salt). Plant growth is demonstrated by increments in projected shoot area (pixels) over time. Plants were imaged 3 days prior to salt application to establish a baseline for growth rate determination. Salt was applied in two equal increments, shown by orange vertical lines, at 31 DAS and 34 DAS and plants were imaged daily until 56 DAS to evaluate the effect of salt application on growth rate. Relative growth rate (RGR) is derived from the difference between the logarithms of the smoothed projected shoot area for 32 DAS and 56 DAS and then dividing by 24. Error bars are s.e.m.

**Figure 3:**
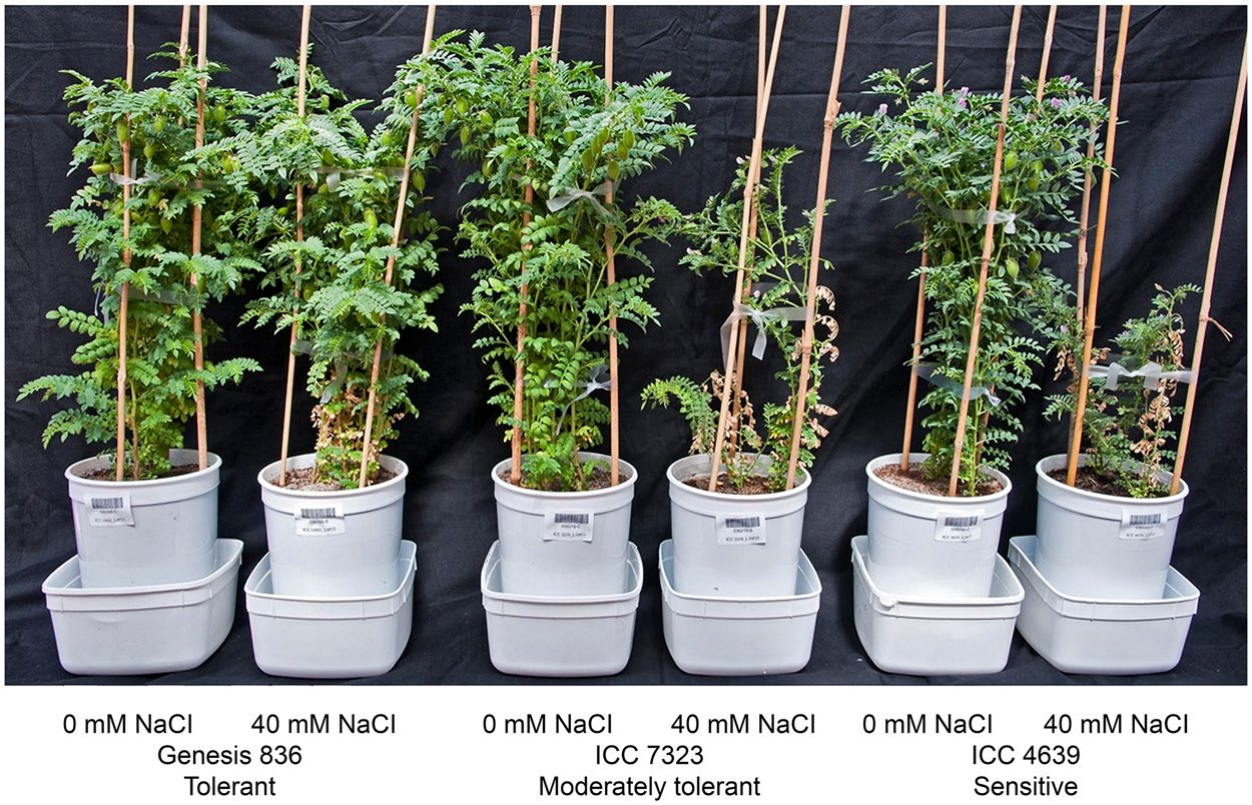
Genotypic variation for salinity tolerance in the chickpea Reference Set. Varying levels of salinity tolerance exhibited by different chickpea genotypes. Exposure of sensitive genotypes to 40 mM NaCl caused severe stunted growth, leaf damage, and led to less number of reproductive sites (flowers and pods) compared to moderately tolerant and tolerant genotypes.

A diversity collection, known as the chickpea Reference Set^26^ (Table S1) was phenotyped under salinity in The Plant Accelerator as shown in Fig. 1 employing a design presented in Figure S1. Broad genetic variation for salinity response exists in the collection (Table 1; Figs 2 and 3), varying significantly between genotypes as evidenced by significant (p ≤ 0.05) genotype-by-treatment interaction for nearly all traits (Table 1).

**Table 1:** Overall values of mean, minimum, maximum, and P-value for effects of genotypes (G), treatments (T) and genotype by treatment interaction (G × T) from the chickpea Reference Set in the glasshouse under salt and control conditions.

Traits	Treatment	MEAN	MIN	MAX	G	T	G × T
Seed yield (g)	Control	4.01	1.06	9.38			
	Salt	2.74	0.00	7.02	n.a	n.a	0.019
Seed number	Control	22.55	1.05	51.01			
	Salt	18.88	0.00	48.65	n.a	n.a	0.006
Shoot biomass (g)	Control	6.36	0.69	12.08			
	Salt	4.60	0.00	10.07	<0.001	<0.001	0.196
Total pod number	Control	26.04	0.00	83.29			
	Salt	23.60	0.18	55.99	n.a	n.a	<0.001
Filled pod number	Control	18.80	0.74	40.61			
	Salt	16.17	0.00	42.63	n.a	n.a	0.004
Empty pod number	Control	7.24	0.00	64.08			
	Salt	7.43	0.00	32.25	n.a	n.a	0.034
100- seed weight (g)	Control	17.84	4.14	35.73			
	Salt	13.10	0.00	29.15	n.a	n.a	<0.001
Plant height (cm)	Control	43.54	23.11	70.18			
	Salt	37.08	12.19	56.20	<0.001	<0.001	0.900
Leaf senescence score	Control	1.45	0.27	10.05			
	Salt	4.53	0.73	10.05	n.a	n.a	<0.001
RGR 32–56	Control	0.05	0.02	0.07			
	Salt	0.04	0.00	0.06	<0.001	<0.001	0.326
Days to flower	Control	67.40	47.90	93.40			
	Salt	68.90	48.50	92.40	n.a	n.a	0.025
Sodium (Na) ions (µmol/gDW)	Control	32.90	0.28	92.79			
	Salt	99.62	10.03	394.36	n.a	n.a	0.006
Potassium (K) ions (µmol/gDW)	Control	969.33	296.00	1743.00			
	Salt	1210.03	347.00	2362.00	<0.001	<0.001	0.970
K:Na	Control	121.23	7.29	3532.14			
	Salt	21.32	3.42	97.13	n.a	n.a	0.034

All measurements are on a pot basis. Number of observation ranged from 201–244. P-values that are not applicable because G × T is significant are indicated by n.a.

### Validation of methodology

To evaluate the suitability of the methodology utilised in this experiment, two genotypes in the Reference Set previously shown to contrast for salinity tolerance were evaluated. The genotypes were ICC 95; highly tolerant to 80 mM NaCl^9^ and ICC 2720; highly sensitive to 100 mM NaCl^33^. In our experiment, ICC 95 and ICC 2720 were shown to greatly differ in their response to salinity, both in terms of growth rate (leaf expansion rate over time) and seed yield. At 53 DAS (21 days after salt application) the significant effect of salinity, manifested by stunted growth, was first seen in ICC 2720, with growth reduction as early as 35 DAS (3 days after salt application). Consequently, ICC 2720 experienced a 50% growth reduction under salinity compared to ICC 95 (Fig. 2). Additionally, these two genotypes differed in their ability to maintain seed yield under salinity. There was a 25% and 80% reduction in seed yield due to salinity in ICC 95 and ICC 2720, respectively (Figure S2).

To further validate the methodology used in the glasshouse experiment, measurements of days to flower, plant height, 100-seed weight, seed number and seed yield from Turretfield field site (pH 6.9 and electrical conductivity (EC_1:5_) 151 ± 20 µS/cm), and Snowtown field site (pH 7.4 and EC_1:5_ ranging from 406 µS/cm to 173 µS/cm at the start and at the end of the trial, respectively), were established with the same measurements in the glasshouse under non-saline conditions. Over 50% of phenotypic variation for plant height, days to flower, 100-seed weight, and seed number could be attributed to genetic variation (Table 2). There was a strong positive correlation of r = 0.72–r = 0.74 for 100-seed weight, and moderate correlations of r = 0.46, r = 0.49, and r = 0.24–0.34 for plant height, days to flower and seed number, respectively between the two field sites and the glasshouse. All these relationships were highly significant (p < 0.001) (Table 2).

**Table 2:** Broad-sense heritability (H2) and pairwise correlations between glasshouse and two field sites for seed number, 100-seed weight, days to flower and plant height.

Traits	Heritability (%)	Site	Glasshouse	Snowtown
Seed number	61	Snowtown	0.24***	
		Turretfield	0.34***	0.48***
100-seed weight	93	Snowtown	0.74***	
		Turretfield	0.72***	0.97***
Days to flower	65	Turretfield	0.49***	
Plant height	61	Turretfield	0.46***	

Level of significance (***p<0.001).

### Large effect of salt on plant measurements in the glasshouse

A significant genotype-by-treatment interaction (p ≤ 0.05) was observed for nearly all traits except for leaf potassium content, plant height, RGR and shoot biomass (Table 1). In cases where genotype-by-treatment interaction was not significant, there was significant genotype variation (p < 0.001) and a significant treatment effect (p < 0.001). Generally, plant growth was negatively impacted by salinity, with plants under saline conditions growing 20% slower compared to plants under non-saline conditions (Table 1; Figure S3). Salinity had a more detrimental effect on growth rate of ICC 2720 compared to ICC 95 (Fig. 2), two genotypes previously reported to contrast for salinity tolerance.

On average salinity reduced shoot biomass and plant height at maturity by 28% and 15%, respectively, compared to non-saline condition (Table 1). Plants grown under saline conditions had greater leaf tissue damage, evidenced by 68% more leaf chlorosis and necrosis in these plants, compared to plants under non-saline conditions (Table 1). Salinity delayed the first appearance of flowers by two days. Plants under non-saline conditions flowered on average at 67 DAS while plants under salinity treatment flowered on average at 69 DAS (Table 1).

The number of total pods and filled pods was negatively impacted under salinity, with plants grown under salt treatment recording a reduction of 9% and 14% in number of pods and filled pods, respectively compared to plants under non-saline conditions (Table 1). On average, the number of empty pods following salt treatment was only slightly increased by 2% (Table 1). Seed number and 100-seed weight (proxy for seed size) were significantly reduced by salt treatment by 16% and 26%, respectively (Table 1). Consequently, seed yield under saline conditions was reduced by 32% relative to non-saline conditions (Table 1).

Plants grown under saline conditions had more Na^+^ in the youngest fully expanded leaf tissues compared to plants grown under non-saline conditions (Table 1). Salt treated plants accumulated 67% more Na^+^ compared to plants under non-saline conditions (Table 1). The range of Na^+^ accumulation in plants under saline treatment ranged from 10 µmol/g DW to 394 µmol/g DW (Table 1) with less than 10% of the genotypes accumulating more than 200 µmol/g DW. A significant genotype-by-treatment interaction (p < 0.001) was observed for Na^+^ and K:Na. Although, the genotype-by-treatment interaction (p = 0.970) was not significant for K^+^, a significant genotype variation (p < 0.001) and a significant difference between the treatments (p < 0.001) was observed (Table 1).

### Relationship between traits in the glasshouse

#### Pearson’s correlation analysis

**Figure 4:**
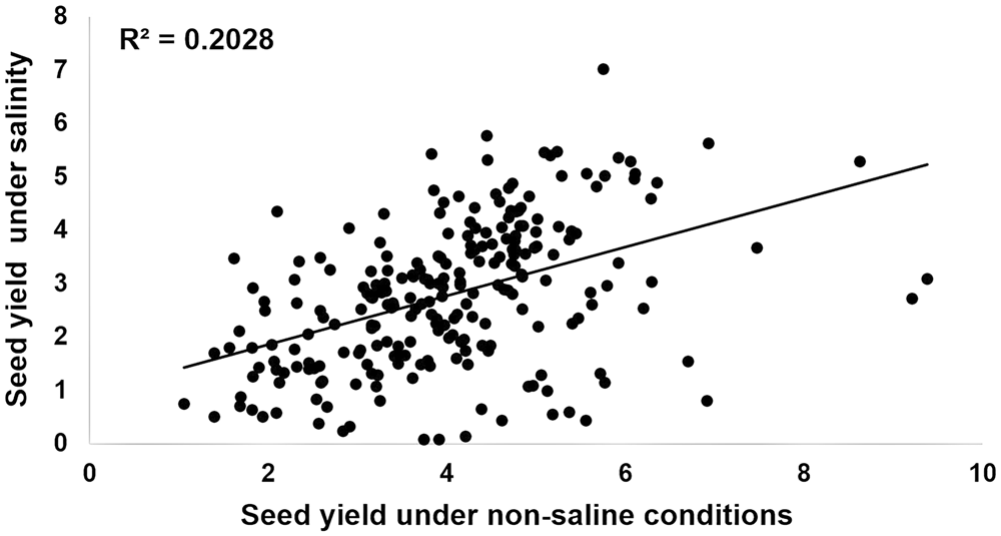
Relationship between seed yield in non-saline and seed yield under salinity. Level of significance **p < 0.01.

Pearson’s correlation analysis was performed to examine the relationship between different traits and seed yield. Seed yield under salinity had a moderate positive correlation with seed yield under non-saline conditions (R^2^ = 0.20), a relationship that was significant (Fig. 4) and confirms that yield potential explained 20% of seed yield under salinity. Hence, in this study, salinity tolerance is defined as the ratio of seed yield under salinity over seed yield under non-saline conditions (seed yield salt/seed yield control). To examine the relationship between traits, ratios of individual traits under saline and non-saline conditions were used. Salinity tolerance was strongly associated with seed number, total number of pods, number of filled pods and harvest index (Table 3; Figure S4). Seed number and number of filled pods accounted for 86% and 79%, respectively, of the variation in salinity tolerance while total number of pods accounted for 70% of the variation in salinity tolerance (Figure S4), harvest index accounted for 68% of this variation (Figure S4). RGR for the entire imaging period (r = 0.33), shoot biomass (r = 0.67), plant height (r = 0.49) and 100-seed weight (r = 0.56) had moderate correlation with salinity tolerance while RGR for the period 41–50 DAS (r = 0.23) was weakly but significantly correlated with salinity tolerance (Table 3). RGR for the period 32–40 DAS (r = 0.09) was not significantly correlated with seed yield (Table 3). Flowering time (r = 0.08) did not play a role in seed yield determination in this study as it had a weak and non-significant relationship with salinity tolerance (Table 3).

**Table 3:** Relationship between traits, salt relative to control (salt/control), determined by correlation analysis.

Traits	Seed yield	Seed number	Shoot biomass	Total pods	Filled pods	Empty pods	100- seed weight	Plant height	Days to flower	Senescence score	RGR 32–40	RGR 41–50	RGR 32–56
Seed yield	1												
Seed number	0.92***	1											
Shoot biomass	0.67***	0.63***	1										
Total pods	0.81***	0.86***	0.72***	1									
Filled pods	0.90***	0.97***	0.61***	0.87***	1								
Empty pods	0.22***	0.23***	0.27***	0.44***	0.22***	1							
100- seed weight	0.56***	0.46***	0.50***	0.46***	0.49***	0.15*	1						
Plant height	0.49***	0.48***	0.49***	0.47***	0.47***	0.20**	0.39***	1					
Days to flower	0.08 ns	0.12 ns	0.10 ns	0.13*	0.11 ns	0.14*	0.03 ns	0.07 ns	1				
Senescence score	−0.14*	−0.12*	−0.10 ns	−0.09 ns	−0.10 ns	−0.05 ns	−0.16*	−0.22**	0.06 ns	1			
RGR 32–40	0.09 ns	0.07 ns	0.24***	0.22***	0.08 ns	0.07 ns	0.09 ns	0.12 ns	−0.01 ns	0.01 ns	1		
RGR 41–50	0.23***	0.29***	0.33***	0.28***	0.30***	0.13*	0.21***	0.45***	0.17*	−0.10 ns	0.23***	1	
RGR 32–56	0.33***	0.34***	0.44***	0.39***	0.36***	0.15*	0.25***	0.45***	0.11 ns	−0.03 ns	0.28***	0.20***	1

Highlighted, are moderate to high correlation coefficients. RGR-relative growth rate. Level of significance (***P<0.001, **P<0.01, *P<0.05, ns=non-significant).

To further explore the relationship between traits measured with seed yield, correlation analysis was conducted separately on data obtained from non-saline and saline conditions. Generally, correlations were stronger for traits measured under salinity (Table S2) compared to non-saline conditions (Table S3). Under salinity, seed number (r = 0.83) and number of filled pods (r = 0.86) were strongly correlated with seed yield (Table S2). On the other hand, shoot biomass (r = 0.68), total number of pods (r = 0.66), 100-seed weight (r = 0.50) plant height (r = 0.52) and RGR for the entire imaging period (r = 0.39) had a moderate correlation with seed yield (Table S2). Leaf Na^+^ content had a moderate but significant negative relationship with seed yield (r = −0.3) (Table S4) while leaf K^+^ had a weak relationship with seed yield (r = −0.19) (Table S4). K:Na was moderately but significantly correlated with seed yield (r = 0.29) (Table S4). Na^+^ had a moderately positive correlation with K^+^ (r = 0.52) and a negative correlation with K:Na (r = −0.64) (Table S4).

Seed number (r = 0.75) and number of filled pods (r = 0.80) had a high correlation with seed yield under non-saline conditions while shoot biomass (r = 0.39) and total pods (r = 0.53) had a moderate correlation with seed yield (Table S3). Conversely, 100-seed weight (r = 0.17), plant height (r = 0.21) and RGR for the whole period the plants were imaged for (r = 0.21) were weakly correlated with seed yield under non-saline conditions (Table S3).

### Path analysis

Path analysis, a standardised partial regression coefficient, was performed to decompose correlation coefficients into components of direct and indirect effects and examine the strength of contribution of the different measured traits on seed yield. Under non-saline conditions, the number of filled pods, seed number and 100-seed weight had a moderate direct positive contribution of 0.50, 0.45 and 0.39, respectively, on seed yield while total number of pods had a moderate indirect positive effect of 0.38 and 0.31, respectively, on seed yield through number of filled pods and seed number (Table S5). Likewise, the number of filled pods had a moderate indirect positive effect of 0.43 on seed yield through seed number (Table S5).

Under salinity, the number of filled pods and seed number had a moderate positive direct effect of 0.45 and 0.41, respectively, on seed yield while 100-seed weight had a weak positive direct effect of 0.28 on seed yield (Table S6). While number of total pods had a moderate indirect positive effect of 0.37 and 0.33, respectively, on seed yield through number of filled pods and seed number, filled pods had a moderate indirect positive effect of 0.4 on seed yield through seed number (Table S6).

Salinity tolerance, defined as seed yield under salinity compared to seed yield under non-saline conditions, was directly predominantly influenced by seed number (0.90) and least influenced by senescence score (−0.006) (Table 4). The total number of pods and filled pods had a strong indirect effect of 0.77 and 0.87, respectively, through seed number, whereas RGR (0.31), plant height (0.44), shoot biomass (0.57) and 100-seed weight (0.41) had a moderate indirect effect on salinity tolerance through seed number (Table 4).

**Table 4:** Direct and indirect effects of yield components on salinity tolerance (salt/control), determined by partial least squares algorithm.

Traits	RGR 32–56	Plant height	Shoot biomass	Total pods	Filled pods	Seed number	100-seed weight	Senescence score	Total effects
RGR 32–56	−***0.029***	−0.006	0.050	0.005	−0.034	0.310	0.039	0.000	0.335
Plant height	−0.013	−***0.013***	0.056	0.006	−0.045	0.435	0.060	0.001	0.488
Shoot biomass	−0.013	−0.006	***0.114***	0.009	−0.058	0.568	0.078	0.001	0.692
Total pods	−0.011	−0.006	0.082	***0.012***	−0.083	0.771	0.072	0.001	0.838
Filled pods	−0.010	−0.006	0.069	0.010	−***0.095***	0.874	0.076	0.001	0.919
Seed number	−0.010	−0.006	0.072	0.010	−0.092	***0.899***	0.071	0.001	0.944
100-seed weight	−0.007	−0.005	0.057	0.006	−0.047	0.411	***0.155***	0.001	0.570
Senescence score	0.001	0.003	−0.012	−0.001	0.010	−0.110	−0.025	−***0.006***	−0.140

Values in the main diagonal part (path coefficients) and off-diagonal part of the table represent direct and indirect effects of yield components on salinity tolerance. Total effects, which correspond to correlation coefficients, are derived from summing up direct and indirect effects. Highlighted are direct effects (bold) moderate indirect (underlined), as well as moderate to high total effects (underlined).

**Figure 5:**
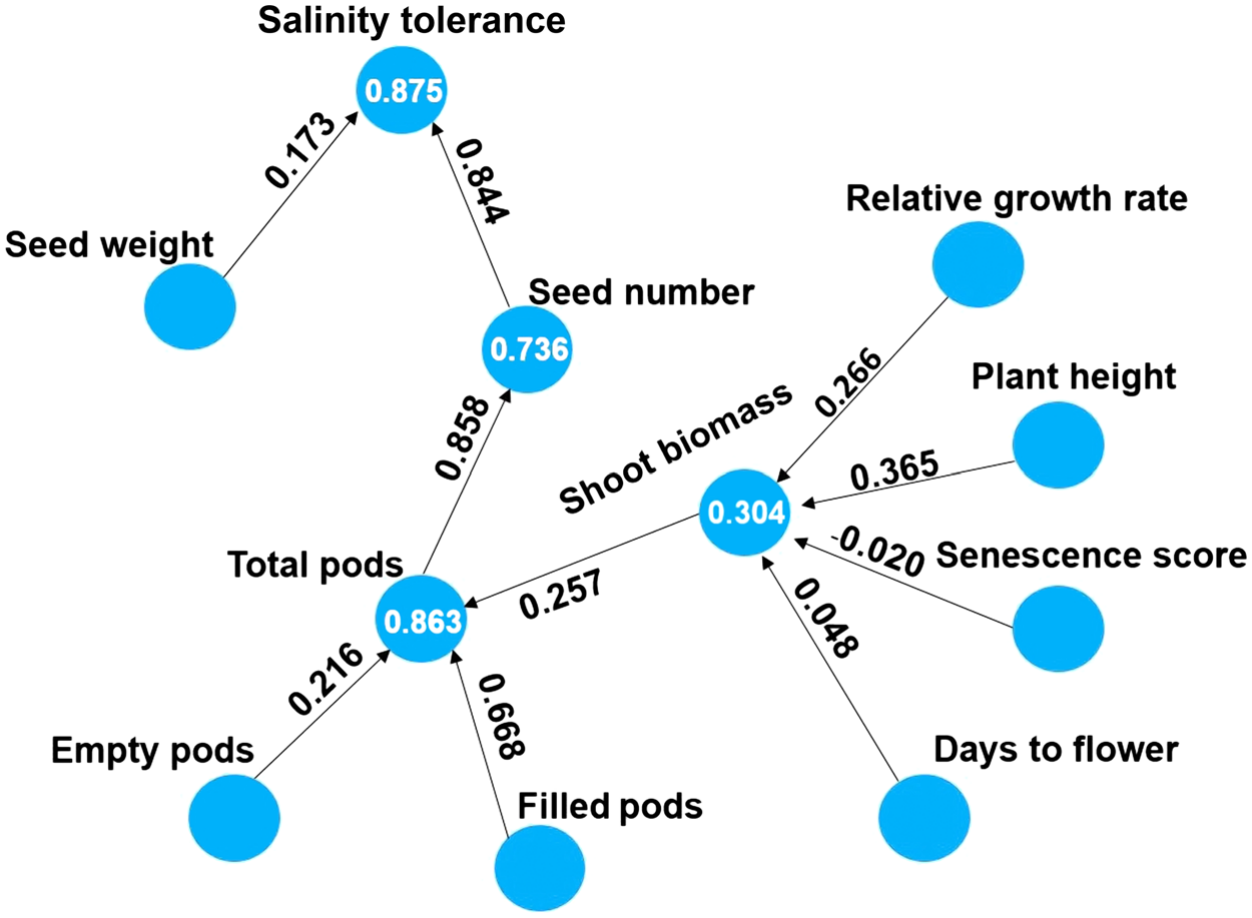
Path analysis diagram of seed yield and yield components. Path analysis derived from structural equation modelling using Partial Least Squares Algorithm method developed by Wold^44^ to demonstrate complex relationship existing between salinity tolerance (seed yield under salt/seed yield under control) and yield related traits. Path coefficients indicated with values on the arrows show direct effect between different yield related traits. Regression coefficients are indicated by values in the circles. Values used for path analysis are relative measurements (salt/control).

A path analysis diagram was used to examine the relationships between salinity tolerance and seed yield components. Relative growth rate (0.27) and plant height (0.37) were found to play a bigger role on shoot biomass than leaf senescence score (−0.02) and days to flowering (0.05) (Fig. 5). Total number of pods was mainly influenced by number of filled pods (0.67), which consequently had a major effect on seed number (0.86), which was the key trait influencing salinity tolerance (0.88) (Fig. 5). Residual variation of only 0.12 was missing from the path diagram developed to determine traits that play direct and indirect role in salinity tolerance determination (Fig. 5). The low residual demonstrates the strength of the model to explain the relationship existing between the traits measured.

## Discussion

Salinity tolerance in higher plants is a complex trait comprised of many sub-traits, suggesting there may be multiple mechanisms and these are likely under the control of many genes^34^. Consequently, efforts to understand the genetic control of salinity tolerance have been challenging. Previous studies have not been able to tease apart the effect of shoot ion independent and shoot ion dependent stress on chickpea growth and yield. In a study of two genotypes contrasting for salinity tolerance (Genesis836 and Rupali), Khan *et al*.^23^ found that osmotic stress imposed in the form of concentrated macronutrient solution, did not have an impact on chickpea biomass. This study looked at osmotic stress, a component of shoot ion independent stress. Other salinity studies in chickpea have shown growth impairment even when toxic ion levels have not been reached in plant tissues including young and old leaves^6–8^. This suggests shoot ion independent stress plays a role in salinity response in chickpea. It is therefore crucial to look at this form of stress, which represents an early response to NaCl exposure.

Image-based phenotyping allows for non-destructive detection of early onset of salinity stress through measurement of plant growth before ions start accumulating in plant tissues. Similar studies have been conducted in barley^32^, and rice^20, 31^. Our study showed that 40 mM NaCl significantly reduced chickpea growth rate with a greater reduction observed in some genotypes. There was broad variation for a range in response time when growth decline was first observed. 20% of the genotypes showed a decline in growth within five days of salt application. For instance, ICC 2720 experienced a significant growth reduction within only three days of salt application, a reduction attributed to shoot ion independent stress (Fig. 2).

Ion regulation plays a major role in salinity tolerance. Leaf senescence/necrosis scores together with measurements of accumulated toxic ions in the shoot can be used to make inferences on salinity tolerance mechanisms^21^. These measures indicate the ability of plants to cope with high salt levels or to reduce salt uptake through the roots and transport to the shoots where significant tissue damage can occur. Maliro *et al*.^22^ scored leaf senescence in a diverse germplasm collection exposed to 60 mM NaCl and identified genotypes with low senescence. However, the genotypes were evaluated for salinity tolerance at the vegetative stage, which does not always translate to tolerance at the reproductive stage^10^. Our study scored leaf senescence at 60 days post salt application and found that some genotypes had started naturally senescing and most scores were confounded by plant age. This partly explains the weak relationship established between leaf senescence and salinity tolerance.

It is thought that Na^+^ but not Cl^−^, is toxic in chickpea^6, 8, 23^. Previous studies have established a negative relationship between Na^+^ content in leaves and seed yield under salinity^6, 8^. We observed a moderate negative correlation of r = −0.3 between seed yield under salinity and Na^+^ content in the youngest fully expanded leaf expressed per unit dry matter (Table S4) with about 10% of the genotypes accumulating more than 200 µmol/g DW. As this study utilised a diverse collection of lines, it was expected that different genotypes may express different salinity tolerance mechanisms, which could explain the moderate correlation. The moderate negative correlation between Na^+^ accumulation and seed yield demonstrates that salinity tolerance in the chickpea Reference Set is partly explained by sodium exclusion. Plants accumulating high Na^+^, generally had more K^+^, an observation also made by Turner *et al*. (2013). Further research is needed to investigate the role of K^+^ and its uptake, efflux, translocation and interaction with Na^+^ during salinity stress in chickpea.

The availability of genetic variation for salinity tolerance is a prerequisite to improve salinity tolerance in chickpea through selection and breeding. Until recently, a lack of streamlined phenotyping facilities has been a bottleneck in studying large diverse collections of chickpea. Previous studies made inferences regarding availability of limited genetic variation for salinity tolerance based on examinations of a relatively small number of chickpea genotypes^24, 25, 35^. However, Vadez *et al*.^7^ demonstrated broad genetic variation for salinity tolerance (measure as seed yield per plant under soil salinity) exists in the chickpea germplasm. In this work we have utilised a high-throughput, non-destructive, efficient, and accurate phenotyping platform to study a diverse collection of chickpea, with the aim of determining traits of relevance for selection in breeding programs. Broad genetic variation for growth rate, plant height, days to flower, leaf senescence, shoot Na^+^ and K^+^ content, shoot biomass, pod number, seed number under salinity and salinity tolerance (measured as seed yield under salinity/seed yield under control) exists in the collection studied here (Table 1).

Salinity had a negative effect on shoot biomass as well as yield and yield components (Table 1). The reduction in seed yield under salinity was attributed to direct reduction in relative growth rate and plant biomass as well as damage to reproductive tissues leading to reductions in number of filled pods, seed number, and 100-seed weight (Table 1). Relative growth rate was only moderately related to shoot biomass at maturity and seed yield. This is because we derived these measurements at the vegetative stage, which emphasizes measurements made at this stage do not always translate to salinity tolerance determined as seed yield under salinity. The number of filled pods and seed number were major determinants of seed yield under salinity, as opposed to 100-seed weight. This is in accordance with previous studies that suggested salinity tolerance in chickpea depends on successful production of reproductive structures under salinity but not the ability to fill seeds^7, 9^. Phenotyping platform development to additionally measure traits such as flower and pod number would assist in the analysis of genetic variation for salinity tolerance in chickpea. Surprisingly, compared to performance under non-saline conditions, 24 genotypes performed better (10% more yield) under the salinity stress imposed in this experiment. This indicates that 40 mM NaCl was perceived as moderate stress by tolerant genotypes, which utilised sodium as an inexpensive osmoticum to stimulate growth and subsequently yield. This phenomenon has previously been observed by Abideen *et al*.^36^ in *Phragmites karka*, a potential bioenergy crop. Moderate salinity treatments have also been shown to stimulate flower production in chickpea^5^ which could ultimately be advantageous to tolerant genotypes.

Correlation and path analysis create an understanding of the relationship between traits. This study showed the role of seed number as the major determinant of improved performance under salinity (Table 3; Table 4; Fig. 5), in line with previous studies by Krishnamurthy *et al*.^9^, Vadez *et al*.^7^ and Vadez *et al*.^10^. However, in contrast to the earlier studies, the phenotyping platform used here allowed us to decompose the correlation analysis into path coefficients to quantify the direct effect of seed number on salinity tolerance. The importance of seed number was masked when performing path analysis on data from either non-saline or salinity conditions. Hence, this points to the importance of defining salinity tolerance and removing the confounding effect of yield potential. Flowering time had no correlation with salinity tolerance (Table 3), an observation also made by other studies evaluating the chickpea Reference Set^6, 9^. This observation resulted from the plants being grown under optimised conditions with adequate water and nutrients ensuring that late flowering genotypes had sufficient time to complete their growth cycles.

Field phenotyping is needed to complement findings from controlled environments to a breeding and agronomic context^37^. However, phenotyping under field conditions is challenging due to the spatial and temporal variability of salinity in the soil profile and the restriction of trials to only one to two periods per year. The variability in stress was evident in the Snowtown field site where moderate salt levels in the soil gradually reduced with season progression possibly due to the dropping of the water table. Carefully designed pot experiments under controlled environments can help identify traits of importance^38^. A relationship was established between data obtained from the glasshouse under non-saline conditions with data from two field sites (typical field site and moderately saline- low salinity field site). Notably, a large proportion of phenotypic variation for all traits measured under both field and control conditions could be attributed to genetic variation. The two field sites had very strong correlation with each other as well as with the glasshouse for 100-seed weight. Moderate but significant correlation was observed between the glasshouse and the two field sites for plant height, days to flower and seed number. This validates the phenotyping methodology used in the glasshouse. However, there is a need to evaluate the chickpea Reference Set in a saline field environment to substantiate the results reported here.

## Conclusion

Image-based phenotyping is a reliable platform for exploring genetic variation for salinity response in chickpea. The methodology used here, coupled with phenotyping platform development through implementation of algorithms to recognise and quantify pod number on plants, can be used to efficiently screen large numbers of accessions. Salt tolerant plants had the ability to maintain growth, successfully produce reproductive tissues, and maintain low levels of Na^+^ in young leaves under salt stress. The study has demonstrated that chickpea is affected by shoot ion independent and to a small extent shoot ion dependent stress and hence there is a need to identify genomic regions that could contribute loci enabling chickpea to withstand the two phases of salinity stress. Seed number was found to be a major contributor to seed yield under salinity and therefore an important selection trait for breeding chickpea cultivars with improved tolerance. Phenotypic data collected from this study can now be linked with genotypic data from all genotypes to conduct genome-wide association mapping with the aim of identifying loci that underlie salinity tolerance in chickpea.

## Materials and Methods

### Plant material

Experimental plant material consisted of 245 lines from the chickpea Reference Set^26^ along with two Australian chickpea cultivars, Genesis 836 and Rupali. Out of the 245 lines, 186 lines were of desi type while 59 lines were of kabuli type. 95% of the lines were landraces with the rest being advanced cultivars and breeding lines (Table S1).

### Phenotyping in the glasshouse

The Reference Set, along with Genesis 836 and Rupali, were phenotyped in an experiment carried out from June 2014 to November 2014 in The Plant Accelerator (http://www.plantphenomics.org.au/services/accelerator/) located at the Waite Campus of the University of Adelaide. The Plant Accelerator is a Plexiglas-clad greenhouse system which allows high penetration of natural light. The average light intensity for the duration of the experiment was 152 µmoles/m2/s. Temperature and relative humidity in the glasshouse was controlled and ranged from 22 ± 2 °C and 40% (day) and 15 ± 2 °C and 90% (night), respectively. It was set up in two Smarthouses (separate growth rooms) utilising 24 lanes by 22 positions. Each Smarthouse was divided into six zones/blocks, each comprising 4 lanes by 22 positions. The design employed for the experiment was a split-plot design in which two consecutive carts formed a main plot (Figure S1). The split-plot design assigned genotypes to main plots, the genotypes being unequally replicated 2–3 times. Treatments (non-saline, saline) were randomized to the two subplots (carts) within each main plot. The main plot design was generated using Digger^39^ and the subplot randomization was done using dae^40^, packages for the R statistical computing environment^41^ The experimental layout used is shown in Fig. 1.

Prior to sowing, seeds were pickled with Pickle-T fungicide and 5 seeds sown 2 cm deep in draining pots (19.5 cm height × 14.9 cm diameter) filled with 2.5 kg of 50% (v/v) University of California (UC) mixture (1:1 peat: sand) and 50% (v/v) cocopeat amended with osmocote (pH 7.5; electrical conductivity (EC_1:5_ 603 µS/cm). Rhizobium inoculum (Group N) was added to each planting hole at sowing. For the first 28 days after sowing (DAS), the volume of water in the pots was maintained approximately at 375 mL (field capacity equivalent to 15% (w/w) water content). Plants were uniformly thinned to two plants per pot. To quantify plant growth rate before salt application and to have a baseline for individual plant growth rate, plants were imaged at 28 DAS for three days (prior to 40 mM NaCl application) using a fixed 5 megapixel visible/RGB camera (Basler Pilot piA2400-12gc) with images taken from three different views (from the top and two side views, rotated at 90°). At 31 and 34 DAS, each pot received 0 or 40 mM NaCl (based on pilot study where 40 mM NaCl was sufficient to discriminate between sensitive and tolerant genotypes), equivalent to applying 100 mL of 0 or 150 mM NaCl, respectively. 40 mM NaCl was delivered in two increments through the base of the pots by standing an individual pot in its own square container containing saline solution. Saline solution moved into the soil through capillary action. Pots were watered and maintained at field capacity (15% (w/w), determined gravimetrically) to maintain salt concentration and to avoid salt leaching. Plants were imaged for a further 22 days after exposure to salt to quantify growth under saline and non-saline conditions. A total of 28,405 visible light (RGB) images obtained were processed in LemnaGrid (LemnaTec) and plant pixels used to compute projected shoot area. Cubic smoothing splines were fitted for each cart to the projected shoot areas for the observed days after sowing using the function smooth.splines in the R statistical computing environment with df set to 5. Relative growth rates (RGR) were computed from the smoothed projected shoot area for each cart for each day of imaging, as described by^42^. It was calculated as the difference in the logarithms of the smoothed projected shoot area for two consecutive days of imaging, which is then divided by the number of days between the imagings. Also calculated was the RGR for the interval 32–56 DAS by taking the difference between the logarithms of the smoothed projected shoot area for 32 DAS and 56 DAS and then dividing by 24.

In addition to data extracted from high-resolution imaging, visual measurements of flowering time (day to first flower) and leaf chlorosis and necrosis on a scale of 1 (healthy) - 9 (dead) according to Maliro *et al*.^22^, were also taken. Other traits measured included leaf sodium (Na^+^) and potassium (K^+^) ion content, plant height, yield and yield components including shoot biomass, seed number, total pod number, empty pod number, filled pod number and 100-seed weight.

### Sodium (Na^+^) and potassium (K^+^) ion content determination

At the podding stage, a single sample of the youngest fully expanded leaf was collected from each pot. Samples were oven dried at 60 °C for 48 hours. Leaf samples were weighed and extracted in 2 mL of 1% (w/w) nitric acid (70% [w/w] Nitric Acid; Chem-Supply NA001-500M, Gillman) at 70 °C for 24 hours, then analysed for Na^+^ and K^+^ content using flame photometry (Model 420 Flame Photometer, Sherwood Scientific).

### Phenotyping in the field

The Reference Set, along with some extra genotypes, was evaluated at two field sites, Turretfield in 2013 and Snowtown in 2014, located in the mid-North of South Australia. Soil cores up to a depth of 20 cm were used to establish pH and electrical conductivity of the soil solution (EC_1:5_) of the two sites. At Turretfield, a randomized complete block design with three replicates was used to assign the 255 genotypes to plots that consisted of 1 m paired rows. At Snowtown, the randomized complete block design had four replicates of 250 genotypes assigned to plots measuring 5 m by 4 m. Prior to sowing, seed was pickled with Pickle T fungicide and Rhizobium inoculum (Group N) applied to sowing furrows. Data including, days to flower, plant height at maturity, seed number, and 100-seed weight were collected.

### Data analysis

Linear mixed models employed in GenStat 17^th^ Edition software were used to analyse a trait and to calculate Best Linear Unbiased Estimates (BLUE) for each genotype. The model for a trait from the glasshouse experiment was:

$${\bf{y}}=X{\boldsymbol{\beta }}+{\bf{Zu}}+{\bf{e}}$$

where **y** is the response vector of values for the trait being analysed; **β** is the vector of fixed effects; **u** is the vector of random effects; and **e** is the vector of residual effects. **X**, and **Z** are the design matrices corresponding to **β** and **u**, respectively. The fixed effect vector, **β′**, is partitioned as follows: $$[\begin{array}{cccc}\mu  & ({{\boldsymbol{\beta }}}_{246\times 1}^{{\rm{G}}})^{\prime}  & ({{\boldsymbol{\beta }}}_{2\times 1}^{{\rm{T}}})^{\prime}  & ({{\boldsymbol{\beta }}}_{492\times 1}^{G:T})^{\prime} \end{array}]$$, where *μ* is the overall mean and the **β**s are the vectors of, Genotype main effects, Treatment main effects and Genotypes-by-Treatment interaction effects, respectively. Also, the random effects vector, **u′** is partitioned as follows: $$[\begin{array}{ccc}({{\bf{u}}}_{2\times 1}^{{\rm{S}}})^{\prime}  & ({{\bf{u}}}_{12\times 1}^{S:Z})^{\prime}  & ({{\bf{u}}}_{528\times 1}^{S:Z:M})^{\prime} \end{array}]$$, where the **u**s are the vectors of, 2 Smarthouse random effects, 6 Zone random effects for each Smarthouse and 44 Main-plot random effects within each Zone within each Smarthouse, respectively. The design matrices **X** and **Z** are partitioned to conform to the partitioning of **β** and **u**, respectively. It is assumed that each subvector of random effects, **u** ^*i*^, is distributed ${N}({{\bf{0}}}_{m},{\sigma }_{i}^{2}{{\bf{I}}}_{m})$, where **0**_*m*_ is the *m*-vector of zeroes, ${\sigma }_{i}^{2}$ is the variance of the *i*th set of random effects, **I**_*m*_ is the identity matrix of order *m*, and *m* is the order of **u** ^*i*^. Further, residual effects **e** are assumed to be $N({{\bf{0}}}_{1056},{\sigma }^{2}\otimes {{\bf{I}}}_{1056})$, where *σ* ^2^ is the variance of individual plants after all other effects have been taken into account.

For the field studies the same general form of mixed model was used, but with the fixed-effect vector partitioned as follows: $$[\begin{array}{ccccc}\mu  & {\beta }^{{\rm{R}}} & {\beta }^{{\rm{C}}} & ({{\boldsymbol{\beta }}}_{b\times 1}^{{\rm{B}}})^{\prime}  & ({{\boldsymbol{\beta }}}_{g\times 1}^{{\rm{G}}})^{\prime} \end{array}]$$ where *μ* is the overall mean, *β* ^R^ and *β* ^C^ are the linear coefficients for Rows and Columns, and the **β**s are the vectors of Block (*b* = 4 or 3) and Genotype (*g* = 255 or 250) main effects, respectively. Also, the random effects vector, **u′**, is partitioned as follows: $$[\begin{array}{cc}({{\bf{u}}}_{r\times 1}^{{\rm{R}}})^{\prime}  & ({{\bf{u}}}_{c\times 1}^{{\rm{C}}})^{\prime} \end{array}]$$, where the **u**s are the vectors of Row (*r* = 12 or 21) and Column (*c* = 87 or 35) random effects, respectively. The residual effects **e** are assumed to be $N({{\bf{0}}}_{n},{\sigma }^{2}{{\boldsymbol{\Sigma }}}_{{\rm{R}}}\otimes {{\boldsymbol{\Sigma }}}_{{\rm{C}}})$, where *σ* ^2^ is the variance of individual plots after all other effects have been taken into account and **Σ**_R_ and **Σ**_C_ are first-order autocorrelation matrices for Rows and Columns, respectively, and *n* = 1020 or 750. Additionally, estimates of broad-sense heritability (H^2^) for traits measured both in the glasshouse and field environments were calculated using the formula derived from^43^. Raw data can be found in Table S7.

### Pearson’s correlation analysis and Path analysis

Pearson’s correlation analysis was conducted in the GenStat 17^th^ edition to establish association between traits. Path analysis was conducted using SmartPLS, software for partial least squares structural equation modelling (PLS-SEM)^44^, with the objective of decomposing correlation coefficients into components of direct and indirect effects to examine the strength of contribution of the different measured traits on seed yield.

## Electronic supplementary material

Supplementary Information

Table S7
