# Supplementary material for: Exploring genetic variation for salinity tolerance in chickpea using image-based phenotyping
Source: Sci Rep. 2017 May 2;7:1300. doi: 10.1038/s41598-017-01211-7 (PMC5430978; doi:10.1038/s41598-017-01211-7)
Supplement: Supplementary file 1 — Supplementary Information [file 41598_2017_1211_MOESM1_ESM.pdf]

Supplementary information

Exploring genetic variation for salinity tolerance in chickpea using image-based phenotyping

Judith Atieno, Yongle Li, Peter Langridge, Kate Dowling, Chris Brien, Bettina Berger, Rajeev K Varshney and Tim Sutton*

Line numbers

grey - lines replicated twice (1 - 213)   green = lines replicated thrice (214 -245)   blue = check lines (246 - 247)

SW Positions

Lanes	23	22	21	20	19	18	17	16	15	14	13	12	11	10	9	8	7	6	5	4	3	2
1	3	3	238	238	213	213	237	237	7	7	2	2	229	229	223	223	20	20	214	214	92	92
2	5	5	210	210	206	206	228	228	18	18	48	48	147	147	165	165	65	65	54	54	76	76
3	140	140	8	8	104	104	234	234	129	129	116	116	161	161	63	63	12	12	159	159	162	162
4	233	233	156	156	199	199	218	218	84	84	176	176	197	197	154	154	24	24	196	196	10	10
5	13	13	45	45	85	85	115	115	215	215	218	218	49	49	193	193	32	32	111	111	64	64
6	29	29	225	225	119	119	79	79	195	195	138	138	66	66	59	59	224	224	51	51	245	245
7	133	133	123	123	139	139	75	75	112	112	53	53	232	232	182	182	44	44	40	40	137	137
8	243	243	226	226	120	120	19	19	21	21	110	110	164	164	55	55	167	167	61	61	174	174
9	108	108	56	56	130	130	189	189	67	67	181	181	239	239	179	179	235	235	102	102	94	94
10	86	86	60	60	217	217	128	128	72	72	225	225	124	124	16	16	42	42	227	227	77	77
11	101	101	201	201	121	121	71	71	117	117	28	28	219	219	114	114	113	113	163	163	9	9
12	151	151	186	186	14	14	214	214	241	241	208	208	160	160	200	200	6	6	173	173	209	209
13	41	41	142	142	222	222	80	80	93	93	43	43	240	240	126	126	217	217	143	143	68	68
14	37	37	33	33	152	152	187	187	246	246	36	36	148	148	204	204	226	226	88	88	103	103
15	223	223	17	17	190	190	183	183	31	31	4	4	146	146	144	144	78	78	231	231	150	150
16	87	87	221	221	134	134	25	25	212	212	131	131	216	216	205	205	50	50	96	96	52	52
17	69	69	82	82	185	185	118	118	236	236	244	244	207	207	180	180	30	30	73	73	22	22
18	198	198	35	35	122	122	39	39	194	194	170	170	27	27	203	203	178	178	175	175	171	171
19	211	211	242	242	246	246	224	224	127	127	141	141	219	219	81	81	125	125	107	107	228	228
20	177	177	157	157	47	47	74	74	220	220	26	26	38	38	191	191	23	23	222	222	91	91
21	1	1	153	153	57	57	34	34	70	70	166	166	149	149	188	188	97	97	98	98	90	90
22	99	99	100	100	106	106	95	95	202	202	220	220	184	184	105	105	158	158	109	109	132	132
23	227	227	89	89	135	135	216	216	230	230	15	15	145	145	247	247	215	215	11	11	58	58
24	46	46	229	229	155	155	168	168	83	83	169	169	172	172	136	136	62	62	221	221	192	192

SE Positions

Lanes	2	3	4	5	6	7	8	9	10	11	12	13	14	15	16	17	18	19	20	21	22	23
1	1	1	234	234	247	247	229	229	48	48	237	237	138	138	160	160	228	228	32	32	9	9
2	37	37	120	120	106	106	239	239	16	16	244	244	176	176	179	179	113	113	40	40	22	22
3	41	41	156	156	142	142	115	115	141	141	110	110	166	166	182	182	20	20	30	30	132	132
4	230	230	47	47	14	14	183	183	216	216	93	93	27	27	167	167	42	42	73	73	150	150
5	13	13	46	46	118	118	212	212	80	80	43	43	242	242	235	235	191	191	88	88	162	162
6	56	56	57	57	95	95	152	152	244	244	84	84	161	161	148	148	196	196	111	111	209	209
7	225	225	153	153	75	75	243	243	185	185	81	81	236	236	105	105	214	214	126	126	137	137
8	5	5	86	86	29	29	202	202	21	21	70	70	197	197	11	11	241	241	92	92	68	68
9	89	89	60	60	139	139	31	31	240	240	146	146	204	204	63	63	59	59	239	239	90	90
10	100	100	3	3	122	122	190	190	72	72	154	154	222	222	200	200	178	178	109	109	64	64
11	241	241	112	112	8	8	220	220	233	233	116	116	53	53	55	55	165	165	77	77	91	91
12	242	242	213	213	177	177	28	28	134	134	232	232	49	49	169	169	107	107	6	6	58	58
13	133	133	45	45	155	155	128	128	135	135	170	170	234	234	203	203	193	193	52	52	143	143
14	99	99	211	211	33	33	18	18	195	195	224	224	235	235	145	145	240	240	62	62	54	54
15	151	151	168	168	71	71	218	218	238	238	149	149	144	144	231	231	24	24	102	102	94	94
16	35	35	108	108	223	223	79	79	7	7	4	4	219	219	180	180	51	51	175	175	158	158
17	157	157	101	101	243	243	181	181	187	187	231	231	38	38	205	205	245	245	12	12	10	10
18	85	85	130	130	186	186	82	82	36	36	83	83	34	34	97	97	65	65	50	50	103	103
19	198	198	201	201	199	199	74	74	129	129	15	15	184	184	232	232	215	215	61	61	247	247
20	210	210	104	104	119	119	189	189	194	194	217	217	208	208	125	125	78	78	171	171	159	159
21	140	140	123	123	206	206	237	237	236	236	127	127	66	66	245	245	188	188	230	230	192	192
22	69	69	233	233	39	39	19	19	67	67	221	221	114	114	44	44	131	131	163	163	174	174
23	87	87	17	17	25	25	227	227	2	2	172	172	117	117	124	124	23	23	98	98	76	76
24	246	246	121	121	238	238	226	226	207	207	147	147	26	26	164	164	96	96	136	136	173	173

Condition numbers: 2-control 1-salt

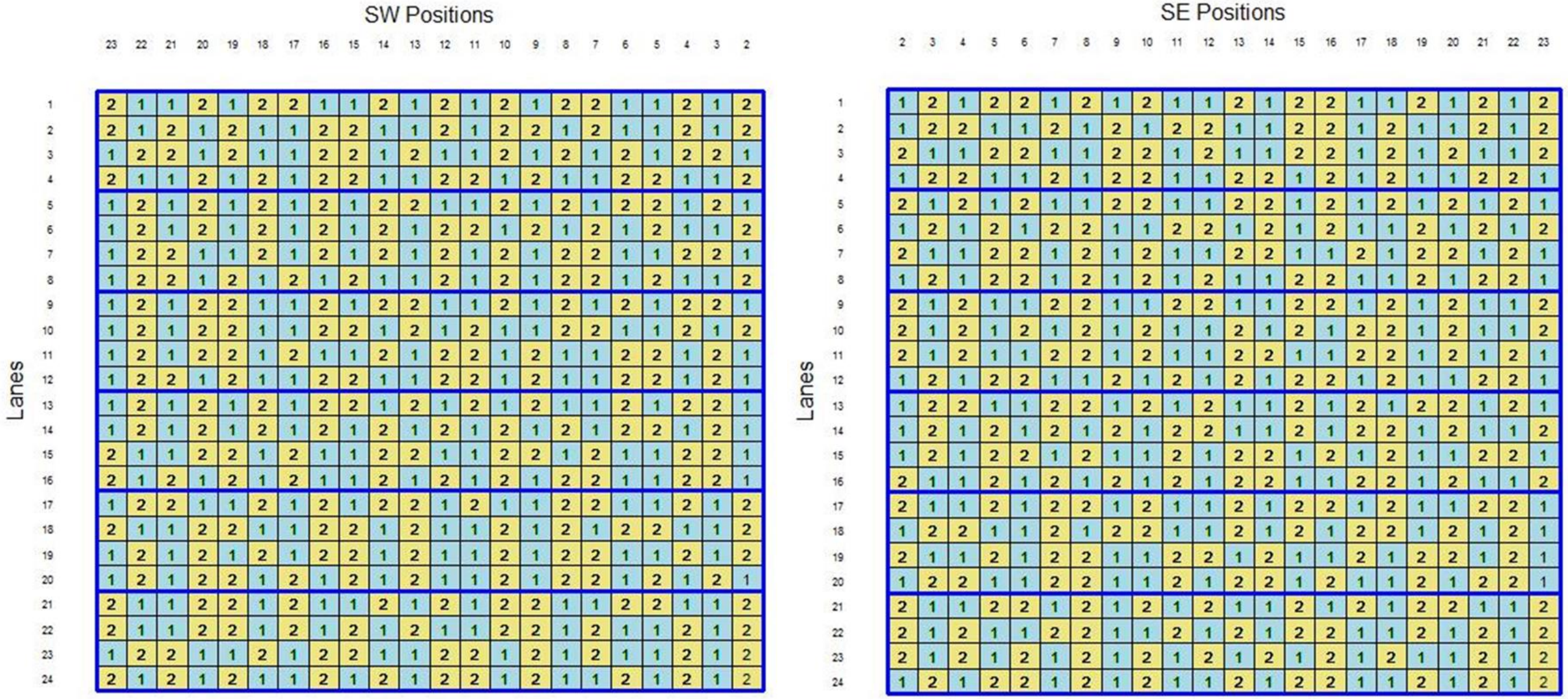

Figure S1: A split-plot design with unequally-replicated, nearly-trend-free main-plot design

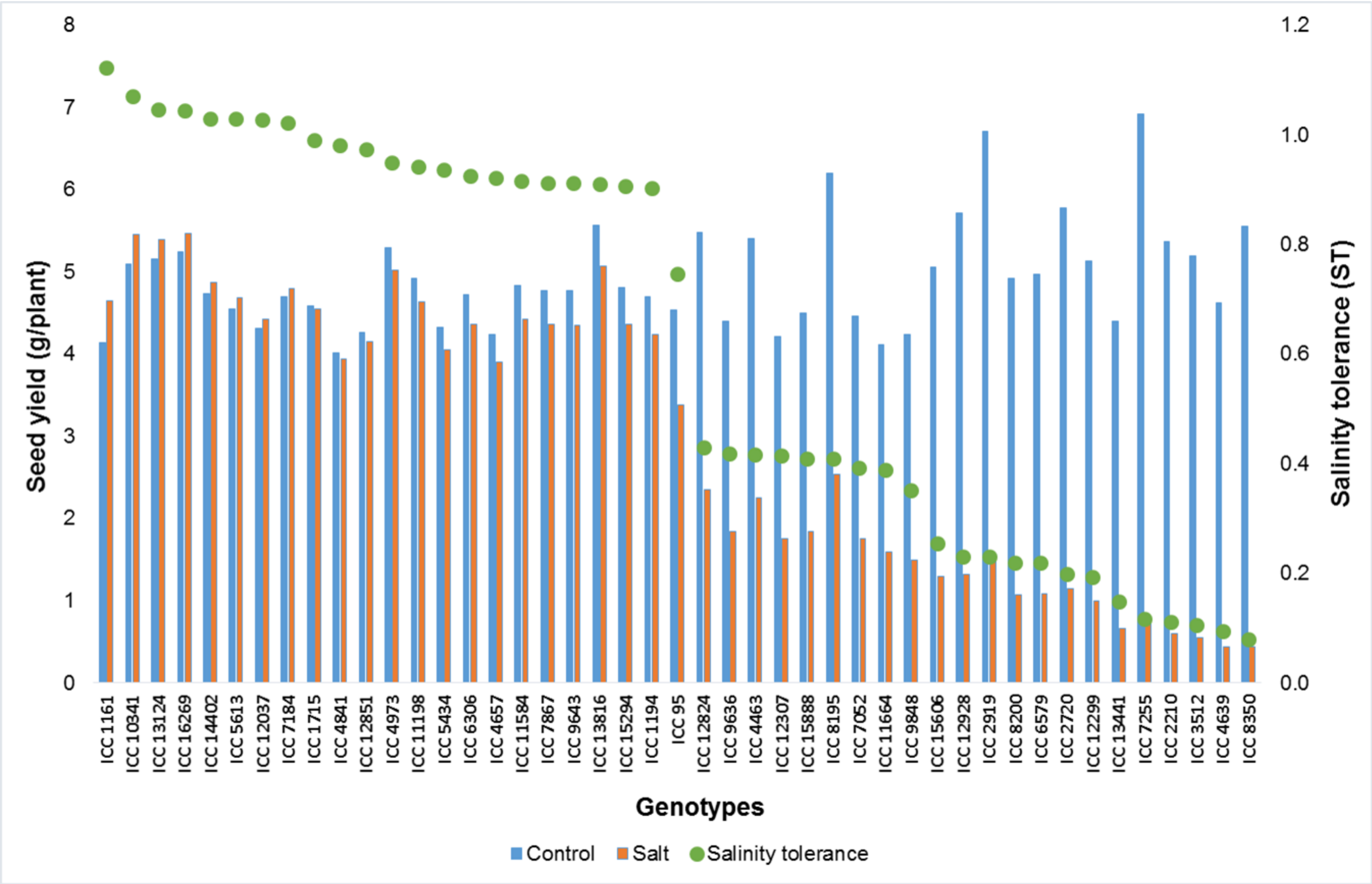

Figure S2: Genotypic variation for salinity tolerance in the chickpea reference set. Genotypes ranking from the most salt tolerant to salt sensitive based on salinity tolerance (seed yield under salinity/seed yield under non-saline conditions.

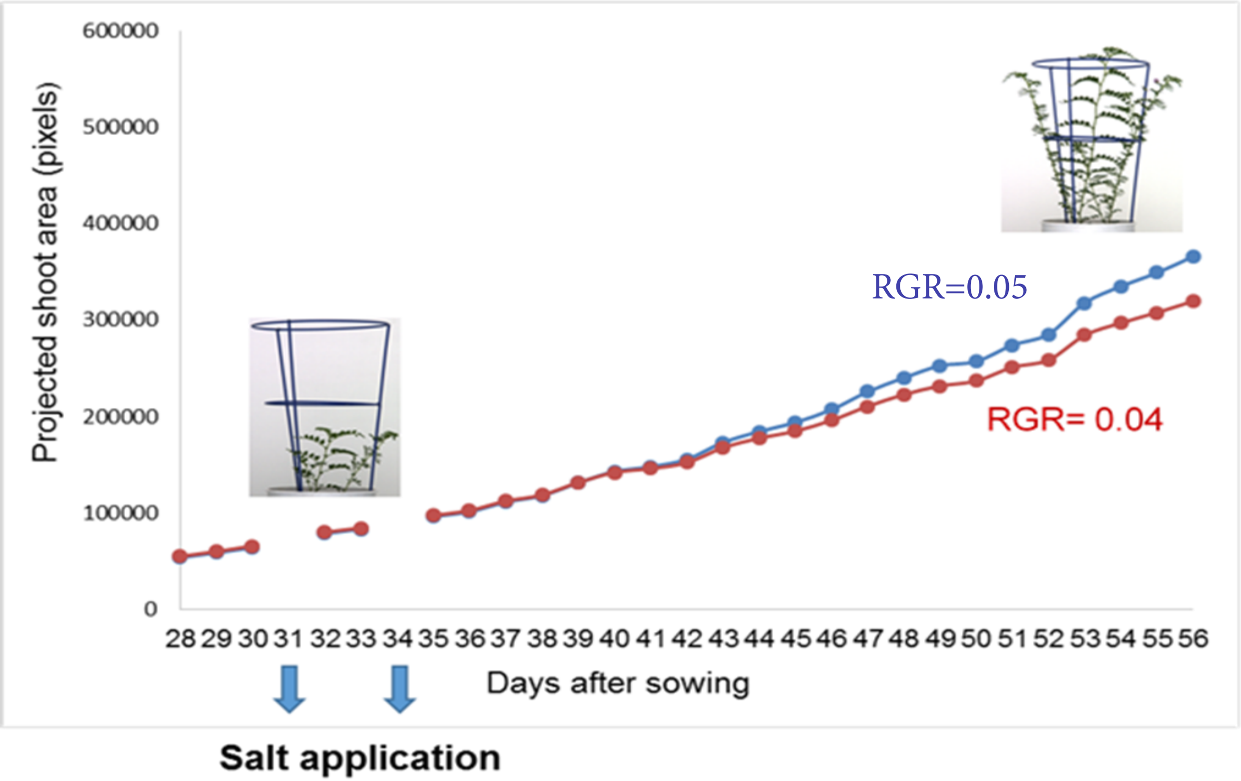

Figure S3: Non-destructive imaging of chickpea plants to determine average growth rate of all genotypes over time under 0 (blue line) and 40 mM NaCl (red line). Plant growth is demonstrated by increments in projected shoot area (pixels) over time. Salt application was done in two equal increments at 31 DAS and 34 DAS and the plants were imaged daily up to 56 DAS to evaluate the effect of salt application on growth of the plants. RGR (relative growth rate) was obtained by taking the difference between the logarithms of the smoothed projected shoot area for 32 DAS and 56 DAS and then dividing by 24. Inset pictures show RGB image of a chickpea plant at 31 DAS and 56 DAS.

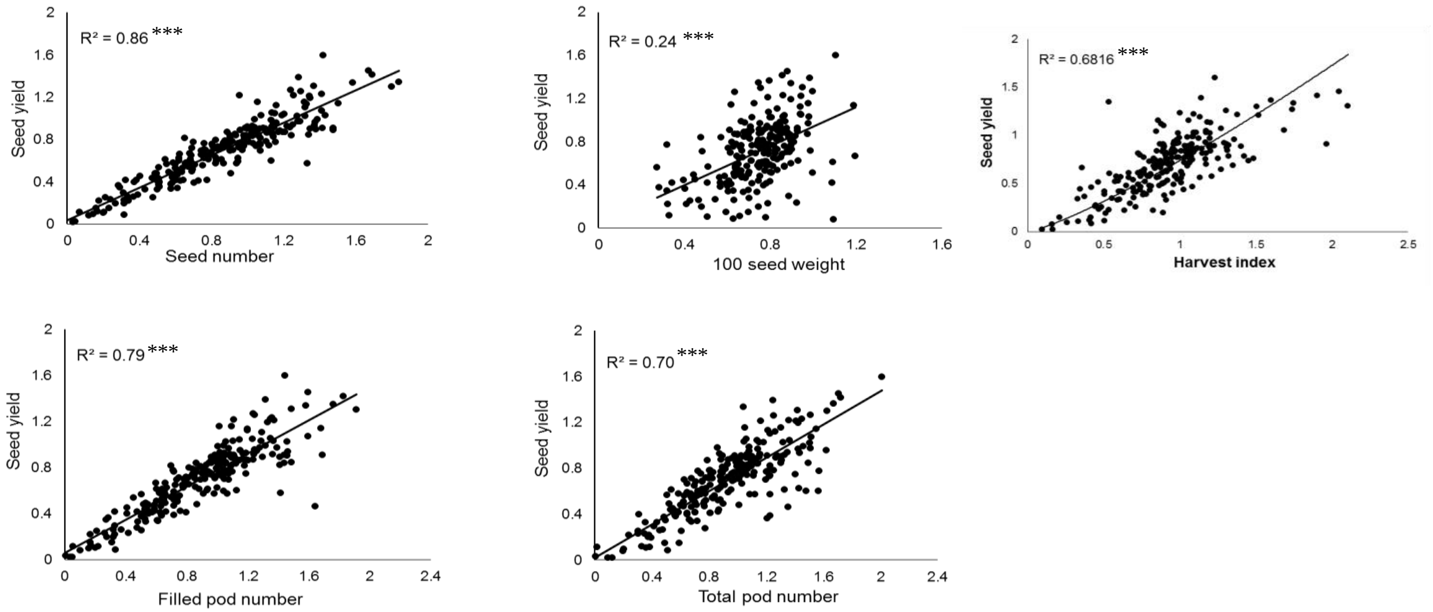

Figure S4: Relationship between seed yield and seed number, 100-seed weight, harvest index, filled pod number and total pod number. These are relative measurements (salt/control) per plant. Level of significance; ***= $p < 0.001$ ** =$p < 0.01$ *= $p < 0.05$

Table S1: Composition of the chickpea reference set

Genotype	Origin	Biological status	Market type
ICC 10018	India	Landrace	Desi
ICC 10341	Turkey	Landrace	Pea-shaped
ICC 10393	India	Landrace	Desi
ICC 10399	India	Landrace	Desi
ICC 1052	Pakistan	Landrace	Desi
ICC 10673	Turkey	Landrace	Desi
ICC 10685	Turkey	Landrace	Desi
ICC 10755	Turkey	Landrace	Kabuli
ICC 1083	Iran	Landrace	Desi
ICC 10885	Ethiopia	Landrace	Kabuli
ICC 10945	India	Landrace	Desi
ICC 1098	Iran	Landrace	Desi
ICC 11121	India	Landrace	Desi
ICC 11198	India	Landrace	Desi
ICC 11279	Pakistan	Landrace	Desi
ICC 11284	USSR	Landrace	Desi
ICC 11303	Chile	Landrace	Kabuli

ICC 11498	India	Breeding material	Desi
ICC 11584	India	Landrace	Desi
ICC 1161	Pakistan	Landrace	Desi
ICC 11627	India	Landrace	Desi
ICC 1164	Nigeria	Landrace	Desi
ICC 11664	India	Landrace	Desi
ICC 11764	Chile	Landrace	Kabuli
ICC 1180	India	Landrace	Desi
ICC 11879	Turkey	Landrace	Kabuli
ICC 11903	Germany	Landrace	Desi
ICC 1194	India	Landrace	Desi
ICC 11944	Nepal	Landrace	Desi
ICC 12028	Mexico	Landrace	Desi
ICC 12037	Mexico	Breeding material	Kabuli
ICC 1205	India	Landrace	Desi
ICC 12155	Bangladesh	Landrace	Desi
ICC 12299	Nepal	Landrace	Desi
ICC 1230	India	Landrace	Desi
ICC 12307	Myanmar	Landrace	Desi
ICC 12328	Cyprus	Landrace	Kabuli

ICC 12379	Iran	Landrace	Desi
ICC 12492	India	Landrace	Kabuli
ICC 12537	Ethiopia	Landrace	Desi
ICC 12654	Ethiopia	Landrace	Desi
ICC 12726	Ethiopia	Landrace	Desi
ICC 12824	Ethiopia	Landrace	Desi
ICC 12851	Ethiopia	Landrace	Desi
ICC 12866	Ethiopia	Landrace	Desi
ICC 12916	India	Landrace	Desi
ICC 12928	India	Landrace	Desi
ICC 12947	India	Landrace	Desi
ICC 13077	India	Landrace	Kabuli
ICC 13124	India	Landrace	Desi
ICC 13187	Iran	Breeding material	Kabuli
ICC 13219	Iran	Landrace	Desi
ICC 13283	Iran	Landrace	Kabuli
ICC 13357	Iran	Landrace	Kabuli
ICC 13441	Iran	Landrace	Kabuli
ICC 13461	Iran	Landrace	Kabuli
ICC 13523	Iran	Landrace	Kabuli

ICC 13524	Iran	Landrace	Desi
ICC 1356	India	Landrace	Desi
ICC 13599	Iran	Landrace	Desi
ICC 13628	Iran	Landrace	Kabuli
ICC 13764	Iran	Landrace	Kabuli
ICC 13816	USSR	Landrace	Kabuli
ICC 13863	Ethiopia	Landrace	Desi
ICC 1392	India	Landrace	Desi
ICC 1397	India	Landrace	Desi
ICC 1398	India	Landrace	Desi
ICC 14051	Ethiopia	Landrace	Desi
ICC 14077	Ethiopia	Landrace	Desi
ICC 14098	Ethiopia	Landrace	Desi
ICC 14199	Mexico	Breeding material	Kabuli
ICC 1431	India	Landrace	Desi
ICC 14402	India	Breeding material	Desi
ICC 14595	India	Landrace	Desi
ICC 14669	India	Landrace	Desi
ICC 14778	India	Landrace	Desi
ICC 14799	India	Landrace	Desi

ICC 14815	India	Landrace	Desi
ICC 14831	India	Landrace	Desi
ICC 1510	India	Landrace	Desi
ICC 15248	Iran	Landrace	Desi
ICC 15294	Iran	Landrace	Desi
ICC 15406	Morocco	Landrace	Kabuli
ICC 15435	Morocco	Landrace	Kabuli
ICC 15510	Morocco	Landrace	Desi
ICC 15518	Morocco	Landrace	Kabuli
ICC 15567	India	Breeding material	Desi
ICC 15606	India	Landrace	Desi
ICC 15610	India	Landrace	Desi
ICC 15612	Tanzania	Landrace	Desi
ICC 15614	Tanzania	Landrace	Desi
ICC 15618	India	Landrace	Desi
ICC 15697	Syria	Landrace	Kabuli
ICC 15762	Syria	Landrace	Desi
ICC 15785	Syria	Landrace	Desi
ICC 15802	Syria	Landrace	Kabuli
ICC 15868	India	Landrace	Desi

ICC 15888	India	Landrace	Pea-shaped
ICC 16207	Myanmar	Landrace	Desi
ICC 16261	Malawi	Landrace	Desi
ICC 16269	Malawi	Landrace	Desi
ICC 16374	Malawi	Breeding material	Desi
ICC 16524	Pakistan	Landrace	Desi
ICC 16654	China	Landrace	Kabuli
ICC 16796	Portugal	Landrace	Kabuli
ICC 16903	India	Landrace	Desi
ICC 16915	India	Landrace	Desi
ICC 1710	India	Landrace	Desi
ICC 1715	India	Landrace	Desi
ICC 1882	India	Landrace	Desi
ICC 1915	India	Landrace	Desi
ICC 1923	India	Landrace	Desi
ICC 2065	India	Landrace	Desi
ICC 2072	India	Landrace	Desi
ICC 2210	Algeria	Landrace	Desi
ICC 2242	India	Landrace	Desi
ICC 2263	Iran	Landrace	Desi

ICC 2277	Iran	Landrace	Kabuli
ICC 2482	Iran	Landrace	Kabuli
ICC 2507	Iran	Landrace	Desi
ICC 2580	Iran	Landrace	Desi
ICC 2593	Iran	Landrace	Kabuli
ICC 2629	Iran	Landrace	Desi
ICC 2720	Iran	Landrace	Desi
ICC 2737	Iran	Landrace	Desi
ICC 283	India	Landrace	Desi
ICC 2884	Iran	Landrace	Desi
ICC 2919	Iran	Landrace	Desi
ICC 2969	Iran	Landrace	Desi
ICC 2990	Iran	Landrace	Desi
ICC 3218	Iran	Landrace	Desi
ICC 3230	Iran	Landrace	Desi
ICC 3239	Iran	Landrace	Desi
ICC 3325	Cyprus	Landrace	Desi
ICC 3362	Iran	Landrace	Desi
ICC 3391	Iran	Landrace	Desi
ICC 3410	Iran	Landrace	Kabuli

ICC 3421	Israel	Landrace	Kabuli
ICC 3512	Iran	Landrace	Desi
ICC 3582	Iran	Landrace	Desi
ICC 3631	Iran	Landrace	Desi
ICC 3761	Iran	Landrace	Desi
ICC 3776	Iran	Landrace	Desi
ICC 3946	Iran	Landrace	Desi
ICC 4093	Iran	Landrace	Desi
ICC 4182	Iran	Landrace	Desi
ICC 4363	Iran	Landrace	Desi
ICC 440	India	Landrace	Desi
ICC 4418	Iran	Landrace	Desi
ICC 4463	Iran	Landrace	Desi
ICC 4495	Turkey	Landrace	Desi
ICC 4533	India	Landrace	Desi
ICC 456	India	Landrace	Desi
ICC 4567	India	Landrace	Desi
ICC 4593	India	Landrace	Desi
ICC 4639	India	Landrace	Desi
ICC 4657	India	Landrace	Desi

ICC 4814	Iran	Landrace	Desi
ICC 4841	Morocco	Landrace	Kabuli
ICC 4872	India	Landrace	Pea-shaped
ICC 4918	India	Advanced cultivar	Desi
ICC 4991	India	Advanced cultivar	Desi
ICC 506	India	Landrace	Desi
ICC 5135	India	Breeding material	Desi
ICC 5221	India	Breeding material	Desi
ICC 5337	India	Landrace	Kabuli
ICC 5383	India	Landrace	Desi
ICC 5434	India	Landrace	Desi
ICC 5504	Mexico	Landrace	Desi
ICC 5613	India	Landrace	Desi
ICC 5639	India	Landrace	Desi
ICC 5845	India	Landrace	Desi
ICC 5878	India	Landrace	Desi
ICC 6263	Union of Soviet Socialist Republics	Landrace	Kabuli
ICC 6279	India	Landrace	Desi

ICC 6293	Italy	Landrace	Desi
ICC 6294	Iran	Advanced cultivar	Desi
ICC 6306	Union of Soviet Socialist Republics	Advanced cultivar	Desi
ICC 637	India	Landrace	Desi
ICC 6537	Iran	Breeding material	Desi
ICC 6571	Iran	Landrace	Desi
ICC 6579	Iran	Landrace	Desi
ICC 67	India	Landrace	Desi
ICC 6802	Iran	Landrace	Desi
ICC 6811	Iran	Landrace	Desi
ICC 6816	Iran	Landrace	Desi
ICC 6874	Iran	Landrace	Desi
ICC 6875	Iran	Landrace	Desi
ICC 6877	Iran	Landrace	Desi
ICC 7052	Iran	Landrace	Desi
ICC 708	India	Landrace	Desi
ICC 7150	Turkey	Landrace	Desi
ICC 7184	Turkey	Landrace	Desi

ICC 7255	India	Landrace	Kabuli
ICC 7272	Algeria	Landrace	Kabuli
ICC 7305	Afghanistan	Landrace	Desi
ICC 7308	Peru	Landrace	Kabuli
ICC 7315	Iran	Landrace	Kabuli
ICC 7323	USSR	Landrace	Pea-shaped
ICC 7413	India	Landrace	Pea-shaped
ICC 7441	India	Landrace	Desi
ICC 7554	Iran	Landrace	Desi
ICC 7571	Israel	Landrace	Kabuli
ICC 762	India	Landrace	Desi
ICC 7668	USSR	Landrace	Kabuli
ICC 7819	Iran	Landrace	Desi
ICC 7867	Iran	Landrace	Desi
ICC 791	India	Landrace	Desi
ICC 8151	USA	Landrace	Kabuli
ICC 8195	Pakistan	Landrace	Desi
ICC 8200	Iran	Landrace	Desi
ICC 8261	Turkey	Landrace	Kabuli
ICC 8318	India	Landrace	Desi

ICC 8350	India	Landrace	Pea-shaped
ICC 8384	India	Landrace	Desi
ICC 8515	Greece	Landrace	Desi
ICC 8522	Italy	Landrace	Desi
ICC 8621	Ethiopia	Landrace	Desi
ICC 867	India	Landrace	Desi
ICC 8718	Afghanistan	Landrace	Desi
ICC 8740	Afghanistan	Landrace	Kabuli
ICC 8752	Afghanistan	Landrace	Kabuli
ICC 8855	Afghanistan	Landrace	Kabuli
ICC 8950	India	Landrace	Desi
ICC 9002	Iran	Landrace	Desi
ICC 9137	Iran	Landrace	Kabuli
ICC 9402	Iran	Landrace	Kabuli
ICC 9434	Iran	Landrace	Kabuli
ICC 95	India	Landrace	Desi
ICC 9586	India	Landrace	Desi
ICC 9590	Egypt	Landrace	Desi
ICC 9636	Afghanistan	Landrace	Desi
ICC 9643	Afghanistan	Landrace	Desi

ICC 9712	Afghanistan	Landrace	Desi
ICC 9755	Afghanistan	Landrace	Desi
ICC 9848	Afghanistan	Landrace	Pea-shaped
ICC 9862	Afghanistan	Landrace	Pea-shaped
ICC 9872	Afghanistan	Landrace	Kabuli
ICC 9895	Afghanistan	Landrace	Pea-shaped
ICC 9942	India	Landrace	Desi

Table S2: **Relationship between traits measured under salinity determined by correlation analysis.** Highlighted, are moderate to high correlation coefficients. Level of significance (***=P<0.001, **=P<0.01, *=P<0.05, ns=non-significant).

Traits	Seed yield	Seed number	Shoot biomass	Total pods	Filled pods	Empty pods	100-seed weight	Plant height	Senescence score	RGR 32-56
Seed yield	1									
Seed number	**0.829*** **	1								
Shoot biomass	**0.679*** **	**0.48*** **	1							
Total pods	**0.657*** **	**0.796*** **	**0.538*** **	1						
Filled pods	**0.86*** **	**0.967*** **	**0.525*** **	**0.817*** **	1					
Empty pods	0.014 ns	0.115*	0.245**	**0.661*** **	0.108 ns	1				
100-seed weight	**0.499*** **	0.11*	**0.642*** **	0.099 ns	0.197*	-0.083 ns	1			
Plant height	**0.517*** **	**0.358*** **	**0.604*** **	**0.459*** **	**0.417*** **	0.247***	**0.463*** **	1		
Senescence score	-0.292**	-0.192*	-0.259**	-0.178*	-0.196*	-0.052 ns	**0.336*** **	**-0.349*** **	1	
RGR 32-56	**0.378*** **	**0.439*** **	**0.323*** **	**0.405*** **	**0.428*** **	0.142*	0.138*	0.258**	-0.179*	1

Table S3: **Relationship between traits measured under non-saline conditions determined by correlation analysis.** Highlighted, are moderate to high correlation coefficients. Level of significance (***=P<0.001, **=P<0.01, *=P<0.05, ns=non-significant).

Traits	Seed yield	Seed number	Shoot biomass	Total pods	Filled pods	Empty pods	100-seed weight	Plant height	Senescence score	RGR 32-56
Seed yield	1									
Seed number	**0.75*** **	1								
Shoot biomass	**0.392*** **	0.119*	1							
Total pods	**0.527*** **	**0.682*** **	0.215**	1						
Filled pods	**0.795*** **	**0.946*** **	0.187*	**0.751*** **	1					
Empty pods	-0.075 ns	-0.011 ns	0.124*	**0.684*** **	0.033 ns	1				
100-seed weight	0.169*	**-0.322*** **	**0.533*** **	-0.223**	-0.213**	-0.103*	1			
Plant height	0.211**	0.054 ns	**0.516*** **	0.199**	0.124*	0.163*	**0.337*** **	1		
Senescence score	0.065 ns	0.002 ns	-0.1*	0.036 ns	0.044 ns	0.001 ns	-0.067 ns	-0.202**	1	
RGR 32-56	0.208**	**0.32*** **	0.14*	0.274**	**0.312*** **	0.067 ns	-0.095 ns	0.051 ns	-0.089 ns	1

Table S4: Relationship between seed yield and sodium and potassium ions under salinity determined by correlation analysis. Highlighted, are moderate to high correlation coefficients. Level of significance (***=P<0.001, **=P<0.01, ns=non-significant).

	Na	K	K:Na	Seed yield
Na	1			
K	**0.52*** **	1		
K:Na	**-0.64*** **	-0.16 ns	1	
Seed yield	**-0.3** **	-0.19 ns	0.29**	1

Table S5: Direct and indirect effects of yield components on seed yield under non-saline conditions determined by partial least squares algorithm. Values in the main diagonal part (path coefficients) and off-diagonal part of the table represent direct and indirect effects of yield components on seed yield. Total effects which corresponds to correlation coefficients is derived from summing up direct and indirect effects. Highlighted are direct effects (bold), moderate indirect effects (underlined) as well as moderate to high total effects (underlined).

Traits	RGR 32-56	Plant height	Shoot biomass	Total pods	Filled pods	Seed number	100-seed weight	Senescence score	Total effects
RGR 32-56	***-0.041***	0	0.013	-0.024	0.157	0.145	-0.034	-0.006	0.208
Plant height	-0.002	***-0.009***	0.047	-0.018	0.062	0.024	0.121	-0.015	0.211
Shoot biomass	-0.006	-0.005	***0.091***	-0.019	0.094	0.054	0.191	-0.007	<u>0.393</u>
Total pods	-0.011	-0.002	0.02	***-0.089***	<u>0.377</u>	<u>0.309</u>	-0.08	0.003	<u>0.526</u>
Filled pods	-0.013	-0.001	0.017	-0.067	***0.502***	<u>0.429</u>	-0.076	0.003	<u>0.794</u>
Seed number	-0.013	0	0.011	-0.061	<u>0.475</u>	***0.453***	-0.115	0	<u>0.749</u>
100-seed weight	0.004	-0.003	0.049	0.02	-0.107	-0.146	***0.358***	-0.005	0.17
Senescence score	0.004	0.002	-0.009	-0.003	0.022	0.001	-0.024	***0.072***	0.064

Table S6: Direct and indirect effects of yield components on seed yield under salinity determined by partial least squares algorithm. Values in the main diagonal part (path coefficients) and off-diagonal part of the table represent direct and indirect effects of yield components on seed yield. Total effects which corresponds to correlation coefficients is derived from summing up direct and indirect effects. Highlighted are direct effects (bold), moderate indirect effects (underlined) as well as moderate to high total effects (underlined).

Traits	RGR 32-56	Plant height	Shoot biomass	Total pods	Filled pods	Seed number	100-seed weight	Senescence score	Total effects
RGR 32-56	***-0.03***	0.013	0.038	-0.059	0.193	0.182	0.039	0.003	<u>0.377</u>
Plant height	-0.01	***0.049***	0.071	-0.067	0.188	0.148	0.129	0.005	<u>0.516</u>
Shoot biomass	-0.01	0.03	***0.118***	-0.079	0.237	0.199	0.179	0.004	<u>0.678</u>
Total pods	-0.01	0.022	0.063	***-0.146***	<u>0.368</u>	<u>0.33</u>	0.028	0.002	<u>0.656</u>
Filled pods	-0.01	0.02	0.062	-0.119	***0.451***	<u>0.4</u>	0.055	0.003	<u>0.859</u>
Seed number	-0.01	0.018	0.057	-0.116	<u>0.436</u>	***0.414***	0.031	0.003	<u>0.828</u>
100-seed weight	-0	0.023	0.076	-0.014	0.089	0.046	***0.279***	0.005	<u>0.498</u>
Senescence score	0.005	-0.017	-0.031	0.026	-0.088	-0.079	-0.094	***-0.014***	-0.292
